# Death after percutaneous dilatational tracheostomy: a systematic review and analysis of risk factors

**DOI:** 10.1186/cc13085

**Published:** 2013-10-29

**Authors:** Marcel Simon, Maria Metschke, Stephan A Braune, Klaus Püschel, Stefan Kluge

**Affiliations:** 1 Department of Intensive Care Medicine, University Medical Center Hamburg-Eppendorf, Martinistr 52, 20246 Hamburg, Germany; 2 Department of Forensic Medicine, University Medical Center Hamburg-Eppendorf, Hamburg, Germany

## Abstract

**Introduction:**

Since the introduction and widespread acceptance of percutaneous techniques in the intensive care unit (ICU) setting, the number of critically ill patients undergoing tracheostomy has steadily increased. However, this procedure can be associated with major complications, including death. The purpose of this study is to estimate the incidence and analyze the causes of lethal complications due to percutaneous dilatational tracheostomy (PDT).

**Methods:**

We analyzed cases of lethal outcome due to complications from PDT including cases published between 1985 and April 2013. A systematic literature search was performed and unpublished cases from our own departmental records were retrospectively analyzed.

**Results:**

A total of 71 cases of lethal outcome following PDT were identified including 68 published cases and 3 of our own patients. The incidence of lethal complications was calculated to be 0.17%. Of the fatal complications, 31.0% occurred during the procedure and 49.3% within seven days of the procedure. The main causes of death were: hemorrhage (38.0%), airway complications (29.6%), tracheal perforation (15.5%), and pneumothorax (5.6%). We found specific risk factors for complications in 73.2% of patients, 25.4% of patients had more than one risk factor. Bronchoscopic guidance was used in only 46.5% of cases.

**Conclusions:**

According to this analysis, PDT-related death occurs in 1 out of 600 patients receiving a PDT. Careful patient selection, bronchoscopic guidance, and securing the tracheal cannula with sutures are likely to reduce complication rates.

## Introduction

Since the introduction and widespread acceptance of percutaneous techniques in the intensive care unit (ICU) setting, the number of critically ill patients undergoing tracheostomy has increased in recent years [1,2]. Given a predicted increase in the numbers of mechanically ventilated patients, a further increase in the number of tracheostomy procedures in the ICU is to be expected [1-4].

The ideal timing of tracheostomy is still a subject of debate as there is no clear evidence that early tracheostomy improves relevant endpoints, such as duration of mechanical ventilation, length of ICU stay, and mortality [5]. Nevertheless, tracheostomy is being undertaken significantly earlier during ICU stay, as the intervention appears to be beneficial in terms of patient comfort, mobility, and reducing the requirement for sedation [1,6]. Percutaneous dilatational tracheostomy (PDT) has gained wide acceptance and has become the procedure of choice for tracheostomy in critically ill patients worldwide [7]. However this procedure, just like surgical tracheostomy, is associated with major complications, including death. It is estimated that each year approximately 500 patients in the United States die or are permanently disabled because of a tracheostomy [8].

Three fatalities due to PDT in our department in recent years prompted us to perform this study. We aimed to analyze cases from the literature and from our own database to determine the incidence of lethal complications due to percutaneous tracheostomy, to reveal the causes of death, to identify risk factors and possible mechanisms for lethal complications, and finally to develop recommendations to further minimize the risks of complication.

## Materials and methods

The study was conducted and reported according to PRISMA guidelines [9].

### Search strategy

A systematic search for articles was performed in PubMed, Embase and the Cochrane Library without restrictions for language searching for case reports, case series, observational studies and randomized trials describing or including fatalities associated with PDT. We focused on studies published from 1985 onward as the percutaneous approach gained popularity after its description by Ciaglia *et al*. [10] in that specific year.

An extensive and sensitive search strategy was chosen using the keywords 'percutaneous tracheostomy’ and 'percutaneous tracheotomy’ to ensure comprehensive retrieval of articles. The search was last updated on 30 April 2013.

### Study selection

Studies were selected independently by two authors (MS and SK). Disagreements between the two authors were resolved by discussion. The titles and abstracts of all publications retrieved by the search strategy were screened for eligibility. Studies on PDT were selected for further evaluation. Full-text review of these studies was performed. Studies were included if one or more PDT-related deaths were described. In addition, the reference sections of all studies on PDT were handsearched for additional relevant publications.

### Data extraction, assessment, completion and synthesis

Data was independently extracted with standardized forms and interpreted by three authors (MS, SB, SK). The results of data extraction were then compared and disagreement resolved by discussion. Included publications were reviewed manually for relevant data. Information concerning patient characteristics (age, gender, main diagnosis, and duration of mechanical ventilation before tracheostomy), tracheostomy procedure (technique, bronchoscopic guidance, pre-interventional ultrasound, performing physician or team, procedure-related difficulties, special circumstances, and risk factors for complications), cause and time of death as well as departmental characteristics (type of ICU, year of introduction of PDT in the department, number of PDTs performed annually and the number of fatalities related to PDT since its introduction in the department) was extracted. If the published data set was incomplete, we contacted the corresponding author via email or post. If the corresponding author did not respond within four weeks, we sent a reminder.

### Retrieval of PDT-related fatalities from our own department

To identify tracheostomy-related fatalities in our own department, all patients who had undergone PDT between 1 January 2005 and 31 December 2012 were identified from the departmental electronic patient database. Cases retrieved from this search were further evaluated for procedure-related deaths by manual analysis of their medical records.

Since 1st January 2005, the Ciaglia Blue Rhino™ technique assisted by bronchoscopic guidance has been the standard technique for PDT in our department. The PDTs were performed at the bedside in one of the 10 departmental ICUs according to a standardized operating procedure [11]. After completion, a protocol documenting key aspects of the procedure was generated and saved in the electronic medical record.

The ethics committee of the Hamburg Chamber of Physicians and the institutional data protection official approved the collection, analysis, and publication of the retrospectively obtained and anonymized data for this noninterventional study.

### Data analysis

Results are presented as medians and ranges or as absolute numbers with percentages. The software used for descriptive analyses was Microsoft Excel 2011 (Microsoft Corp., Redmond, WA, USA).

## Results

### Study and case selection

**Figure 1:**
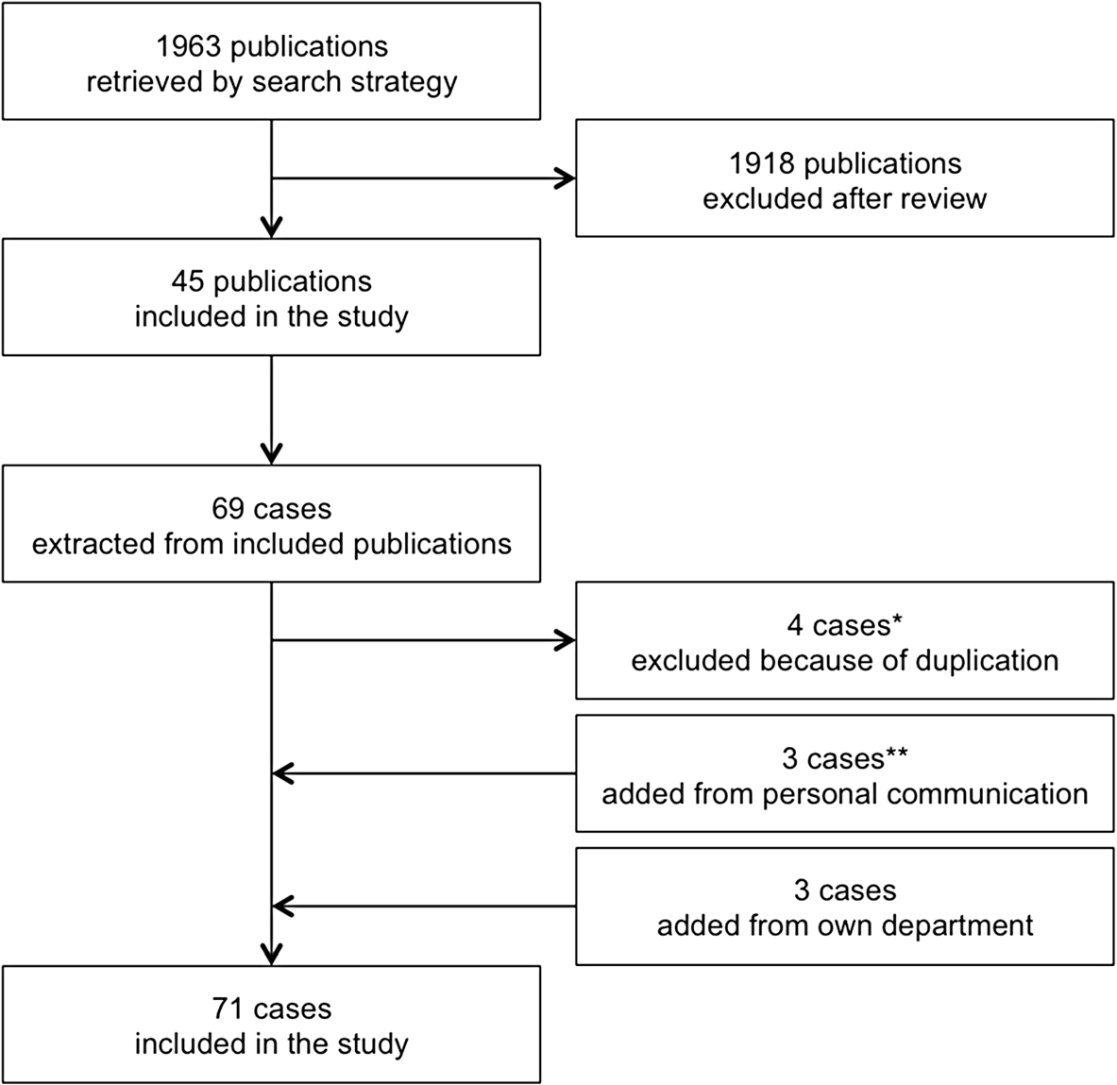
**Process of case selection.** *[12-14]. **[15,16].

The search strategy yielded a total of 1,963 articles. From these publications 45 studies describing 65 PDT-related fatalities were included in the study. Three more cases could be added from personal communication with the corresponding authors. Three additional cases were included from our own departmental records. Overall, 71 cases of PDT-related death were included and analyzed in this study. The process of case selection is summarized in Figure 1.

By contacting the corresponding authors, case specific data could be added in 20 cases and departmental data concerning the total numbers of PDT procedures and PDT-related fatalities could be obtained from 17 departments.

### Study characteristics

We found sixteen case reports or case series, seven retrospective studies, twenty prospective observational studies and two randomized trials. Study characteristics are summarized in Table 1.

**Table 1:** Characteristics of studies included

**Study**								**Departmental data obtained by personal communication with the corresponding author**		
**Author**		**Year**	**Design**	**Population**	**Number of PDTs**	**Number of deaths**	**Mortality rate**	**Number of PDTs**	**Number of deaths**	**Mortality rate**
Byhahn	[16]		CR			2					
Cobean	[15]		CR			1					
Hutchinson	[17]	1991	CR			1					
Cokis	[18]	2000	CR			1					
Hürter	[19]	2000	CR	Surgical		1		252		2	0.79
Drage	[20]	2002	CR			1					
Maeda	[21]	2002	CR	Mixed		1					
Soubirou	[22]	2002	CR	Surgical		1					
Ryan	[23]	2003	CR			1					
Shlugman	[24]	2003	CR	Neurological		1		700		1	0.14
McCormick	[25]	2005	CR	Mixed		3		2,100		4	0.19
Grant	[26]	2006	CR	Mixed		3					
Rosolski	[27]	2006	CR	Mixed		1		420		1	0.24
Ayoub	[28]	2007	CR			1					
Zehlicke	[29]	2007	CR			1					
Wang	[30]	2009	CR			1					
Hoiting	[31]	2010	CR			2					
Gilbey	[32]	2012	CR	Mixed		1		420		1	0.24
Ivatury	[33]	1992	RS		61	1	1.64				
Cobean	[34]	1996	RS	Mixed	65	1	1.54	1,080		2	0.19
van Heurn	[35]	1996	RS	Mixed	150	1	0.67	880		1	0.11
Thompson	[36]	2001	RS	Medical	300	1	0.33				
Pandit	[37]	2006	RS	Mixed	501	1	0.20	1,265		2	0.16
Klein	[38]	2007	RS	Mixed	207	1	0.48				
Dennis	[39]	2013	RS		3162	5	0.16	3,162		5	0.16
Toye	[40]	1986	POS		94	1	1.06				
Marelli	[41]	1990	POS		61	1	1.64				
Wang	[42]	1992	POS	Surgical	7	1	14.29				
Friedman	[43]	1993	POS		100	1	1.00				
Cole	[44]	1994	POS		55	1	1.82				
Barba	[45]	1995	POS	Surgical	27	1	3.70				
Muhl	[46]	1995	POS	Surgical	14	1	7.14	720		1	0.14
Joosten	[47]	1996	POS	Surgical	53	1	1.89	420		1	0.24
Marx	[48]	1996	POS		254	1	0.39				
Berrouschot	[49]	1997	POS	Neurological	76	2	2.63	900		2	0.22
Walz	[50]	1998	POS		337	2	0.59				
Suh	[51]	1999	POS		95	2	2.11	1,040		2	0.19
Escarment	[52]	2000	POS		162	2	1.23				
Kearney	[53]	2000	POS	Mixed	827	5	0.60	5,400		9	0.17
Lim	[54]	2000	POS		261	2	0.77				
Norwood	[55]	2000	POS		422	1	0.24				
Tan	[56]	2004	POS		352	1	0.28				
Byhahn	[57]	2005	POS	Surgical	474	2	0.42	2,775		4	0.14
Chiu	[58]	2005	POS	Medical	107	1	0.93	900		1	0.11
Páez	[59]	2005	POS		38	2	5.26				
Porter	[60]	1999	RT	Surgical	12	1	8.33				
Massick	[61]	2001	RT	Medical	50	1	2.00				
Own cases				Mixed		3		1,873		3	0.16
**Summary**					**8324**	**71**	2.18*	**24,307**		**42**	**0.17**

*Calculated mean mortality rate including data from retrospective studies, prospective observational studies and randomized trials. *CR*, case report, *RS*, retrospective study, *POS*, prospective observational study, *RT*, randomized trial.

### Summary and analysis of all cases

The median age was 66 years (range 4 to 95). For further details concerning patient characteristics see Table 2. Most tracheostomies were performed using the Ciaglia (45.1%) and the Ciaglia Blue Rhino^™^ (26.8%) technique. Bronchoscopic guidance was used in 46.5% of cases, but no cases involved pre- or peri-interventional ultrasound.

**Table 2:** Patient characteristics

**Characteristic**	**Values**	
Number of patients	71	
Median age (years)	66	(range 4-95)
Gender		
female	33	(46.5%)
male	16	(22.5%)
not specified	22	(31.0%)
Main diagnosis		
pulmonary disease	15	(21.1%)
neurologic disease	18	(25.4%)
trauma	9	(12.7%)
cardiac disease	5	(7.0%)
surgical procedure	4	(5.6%)
vascular disease	3	(4.2%)
not specified	17	(23.9%)
Type of intensive care unit		
interdisciplinary	13	(18.3%)
surgical	11	(15.5%)
medical	7	(9.9%)
neurological	6	(8.5%)
cardiothoracic	1	(1.4%)
trauma	3	(4.2%)
not specified	30	(42.3%)
Median duration of intubation before tracheostomy (days)	11	(range 0-33)

The major causes of death were tracheostomy-related hemorrhage in 27 patients (38.0%) and airway complications in 21 patients (29.6%). In 31.0% of cases, fatal complications occurred during the procedure and in 49.3% of cases within seven days of the procedure. 73.2% of patients had specific risk factors and 25.4% of patients had more than one risk factor. For further details about the causes of death and time of complications see Table 3.

**Table 3:** Causes and time of death after PDT

**Cause of death**	**Total number**		**Time of death**			
			**Intra-procedural**		**Post-procedural**	
Total number	71		22	(31.0%)	49	(69.0%)
Hemorrhage	27	(38.0%)	3	(11.1%)	24	(88.9%)
- innominate artery	11	(40.7%)			11	(100.0%)
- aortic arch	2	(7.4%)			2	(100.0%)
- subclavian artery	1	(3.7%)	1	(100.0%)		
- thyroid artery	1	(3.7%)			1	(100.0%)
- other artery	1	(3.7%)	1	(100.0%)		
- venous	5	(18.5%)	1	(20.0%)	4	(80.0%)
- diffuse/unknown	6	(22.2%)			6	(100.0%)
Airway complications	21	(29.6%)	7	(33.3%)	14	(66.7%)
- dislocation of the tracheal cannula	11	(52.4%)	1	(9.1%)	10	(90.9%)
- lost airway during the procedure	4	(19.0%)	3	(75.0%)	1	(25.0%)
- paratracheal misplacement of the tracheal cannula	3	(14.3%)	3	(100.0%)		
- obstruction of tracheal cannula	2	(9.5%)			2	(100.0%)
- hypoxemia during cannula replacement	1	(4.8%)			1	(100.0%)
Tracheal perforation	11	(15.5%)	1	(9.1%)	10	(90.9%)
Pneumothorax	4	(5.6%)	4	(100.0%)		
Bronchospasm	3	(4.2%)	3	(100.0%)		
Cardiac arrest/arrhythmia	3	(4.2%)	3	(100.0%)		
Sepsis	1	(1.4%)			1	(100.0%)
Unknown	1	(1.4%)	1	(100.0%)		

#### ***Hemorrhage***

We found 27 deaths (38.0%) due to hemorrhage [18,19,22-26,28-31,35,37,39,43],[46,49,54,57-59]. The source of bleeding was arterial in 16 cases (59.3%). Most often arterial bleeding originated from tracheovascular fistula formation involving the innominate artery (11 cases). The majority of bleeding incidents (75.0%) occurred between one day and one month after the tracheostomy procedure, with a median of five days. The following known risk factors were retrospectively attributed as potentially relevant in these fatalities secondary to hemorrhagic complications: not using bronchoscopic guidance (eight cases), low tracheostomy site (five cases), coagulopathy (five cases), previous surgery to the neck (four cases), previous radiotherapy (one case), obesity (one case), anatomical abnormality (one case), paratracheal misplacement of the tracheal cannula (one case), malpositioned cannula tip (one case), and high cuff pressure (one case). Twenty-one patients (77.8%) had at least one of the described risk factors.

#### ***Airway complications***

Twenty-one deaths (29.6%) due to airway complications were reported [32,34,38-40,42,44,48,50-53,56],[60,61]. Main causes of death in this group were dislocation of the tracheal cannula (52.4%), lost airway during the procedure (19.0%) and paratracheal misplacement of the tracheal cannula (14.3%). Performing the procedure without bronchoscopic guidance (six cases), by a team relatively inexperienced with the procedure (five cases), in obese patients (eight cases), in patients with a difficult airway (two cases), not securing the tracheal cannula with sutures (three cases), early cannula replacement (one case), and post-procedural care by an inexperienced team (one case) were determined as risk factors for these fatalities attributed to airway complications. Nineteen patients (90.5%) had at least one of the described risk factors.

#### ***Tracheal perforation***

Including our own cases, a total of 11 deaths (15.5%) were due to tracheal perforation [17,20,47,49,53,57,59]. Performing the procedure without using bronchoscopy (five cases), by an inexperienced team (four cases), in an obese patient (one case), in a child (one case), noticing a kinking of the guidewire postprocedurally (two cases), and anatomic abnormalities of the spine (one case) were determined as risk factors for this kind of complication. Nine patients (81.8%) had at least one of the described risk factors.

#### ***Pneumothorax***

Four deaths (5.6%) resulting from a pneumothorax were reported [15,27,36,55]. Two deaths were due to tension pneumothoraces and two were due to bilateral pneumothorax. Primary symptoms were subcutaneous emphysema and/or hypoxemia. Chronic obstructive pulmonary disease (three cases) was stated as underlying condition putting patients at risk for this type of complication.

#### ***Bronchospasm***

Three deaths (4.4%) due to bronchospasm were reported [33,45,53]. Severe acute respiratory distress syndrome (ARDS) (two cases) and chronic obstructive pulmonary disease (one case) were stated as underlying conditions putting patients at risk for this type of complication.

#### ***Cardiac arrest/arrhythmia***

Three intraprocedural deaths (4.4%) due to cardiac arrest were reported [41,50,57]. Cardiac disease and surgery to the heart were recognized as underlying conditions putting patients at risk for this type of complication.

#### ***Sepsis***

One death (1.5%) was due to sepsis [21]. Sepsis was due to mediastinitis originating from the tracheostomy site. Previous sternotomy performed for the treatment of an aneurysm of the thoracic aorta was identified as a risk factor.

### Mortality rate

The mean mortality rate calculated from retrospective studies, prospective observational studies and randomized trials included in this study and overlooking 8,324 PDT procedures was 2.18%.

Departmental data could be obtained from 17 departments responsible for a total number of 24,307 PDT procedures and 42 fatalities. The incidence of death attributable to PDT calculated from these numbers was 0.17%.

In our own department 1,873 patients underwent PDT between 1 January 2005 and 31 December 2012 and three procedure-related fatalities occurred resulting in an incidence of PDT-related lethal complications of 0.16%.

## Discussion

We analyzed 71 cases of death due to PDT. The incidence of death related to PDT calculated from departmental data provided by corresponding authors was 0.17%, which is in line with the lethal complication rate in our own institution. The main reasons for the catastrophic events resulting in death were vascular injuries and airway complications. We found specific risk factors in 73.2% of patients, and 25.4% of patients had more than one risk factor. To the best of our knowledge this is the first systematic analysis of lethal complications due to PDT.

Gilbey *et al*. recently published a case series of fatal cases due to PDT and concluded that this event usually results from vascular injury [32]. However, their report included only seven cases, without providing further information about demographics, PDT technique, use of bronchoscopy or ultrasound.

Even after surgical tracheostomy, fatal complications can occur. In a survey of members of the American Academy of Otolaryngology, Head and Neck Surgery two-thirds of (mainly surgical) tracheostomy-related catastrophic events were reported to be mainly due to loss of airway or bleeding [8].

Tracheostomy-related hemorrhage was the most common cause of death in our study. Massive hemorrhage is a rare but devastating complication after any form of tracheostomy and usually originates from tracheoarterial fistula formation. The majority of cases occur within three days to six weeks of tracheostomy, and risk factors include pressure necrosis from high cuff pressure, mucosal trauma, malpositioned cannula tip, low tracheal incision, excessive neck movement, radiotherapy, or prolonged intubation [26]. We found such risk factors in 77.8% of these patients. In 29.6% of studied patients with fatal bleeding, performing the procedure without bronchoscopy was determined as a risk factor. In this group, placement of tracheostomy was too low in five patients, of which two had an aortic arch laceration. Coagulation dysfunction or platelet dysfunction were present in five patients. These were not deemed to be the primary cause of bleeding in two cases as these patients died from acute and sudden bleeding from specific blood vessels and without diffuse bleeding. Indeed, it has been shown previously, that even in severe thrombocytopenia, PDT can be safely performed after preprocedural correction [62].

A major risk factor seems to be a low tracheal incision, as was the case in five of twenty-seven patients with bleeding complications in our study. The site of puncture should ideally be selected between first and second or second and third tracheal rings [63]. In one study, the site of tracheal puncture was changed in 24% of patients as a result of prior ultrasound [64]. Furthermore, preprocedural ultrasound and clinical examination have been used to detect abnormal pretracheal vascular anatomy. Therefore, several authors recommend ultrasound to improve the safety of PDT [64-67].

The second most frequent cause of death was airway complications. The tracheal cannula was placed outside the tracheal lumen in three cases. Of note, in all of these cases bronchoscopic guidance was not used. Despite the lack of randomized controlled trials many authors think that the use of bronchoscopic guidance significantly increases the safety of PDT as it can guide correct placement of the introducer needle, the guidewire, and the cannula during the procedure [68,69].

In a further 11 patients, the tracheal cannula accidentally dislocated postprocedurally. While the surgical approach allows easy reinsertion of the tracheal cannula, airway complications such as accidental decannulation or tube obstruction are well-described problems of the percutaneous technique [70]. Some researchers have proposed that fixing the tracheal cannula to the skin with sutures for the first postprocedural week may decrease cannula-related complications such as accidental decannulation and postoperative bleeding [71,72].

In two of the eleven deaths (18.2%) due to posterior tracheal wall perforation, kinking of the guidewire was noticed after its removal during the procedure. Other researchers have postulated guidewire kinking as a possible mechanism for perforation of the posterior tracheal wall [73,74]. Thus, kinking of the guidewire must be avoided and its occurrence should raise suspicion of potential injury to the posterior tracheal wall prompting further investigation.

To avoid complications, PDT should only be considered in selected patients without contraindications. Contraindications to PDT include anatomic distortion of the neck, the presence of a difficult airway, severe ARDS, uncorrectable coagulopathy, and the presence of an unstable cervical spine [68]. However, most of these contraindications are relative and also dependent on the skill of the operator. Of the ten cases (14.1%) in our study, where the performing team was deemed to be relatively inexperienced in the procedure, contraindications were also present in two patients (20.0%).

Because of the growing numbers of patients requiring ventilatory support, the frequency of tracheostomy in the ICU has increased over the last decades as has the number of PDT-related publications (Additional file 1). In addition, it is conceivable that after the introduction and widespread acceptance of percutaneous techniques, enthusiasm about the ease of performing this procedure at the bedside may have resulted in relative overuse by intensivists.

A general limitation of this systematic review is the possibility of under- or over-estimating the true incidence of PDT-related death. Accurate risk assessment in clinical medicine is most difficult when an event is rare and available evidence is based on self-reported data [75]. Additionally, concerning the topic of this study, there is sometimes considerable difficulty in differentiating whether an adverse event is due to being critically ill with a tracheostomy or whether it is a complication of the PDT procedure itself. However, in all cases included in this study, the authors of the original publications judged death to be most likely related to the PDT procedure itself. Calculated from departmental data provided by the corresponding authors - where we are confident of both the completeness of reporting and the denominator - the mortality rate from PDT was 0.17%. This is very much in line with the mortality rate of 0.16% reported in the largest study published to date [39] and the mortality rate of 0.16% in our own institution.

In our opinion, this first systemic analysis of the incidence and causes of PDT-related lethal complications adds important evidence to the literature and serves to remind clinicians about potentially life-threatening complications and to help them minimize risk factors by choosing suitable patients and safe procedural strategies.

## Conclusions

In conclusion, PDTs, which are frequently performed in ICUs worldwide, are associated with an average mortality of one in every six hundred procedures. Major risk factors are present in a substantial proportion of these patients. To prevent severe complications, the results of this systematic review, several authors and professional guidelines suggest that the following measures may improve the safety of PDT: strict consideration of contraindications, bronchoscopic guidance during the entire procedure, performance by an experienced team, avoidance of a low tracheostomy puncture site and avoidance of guidewire kinking as well as the use of outer flange tracheal cannula sutures.

## Key messages

• PDT-related death occurs in one out of six hundred procedures.

• Careful patient selection, bronchoscopic guidance, and securing the tracheal cannula with sutures are likely to reduce complication rates.

## Abbreviations

ARDS: Acute respiratory distress syndrome; ICU: Intensive care unit; PDT: Percutaneous dilatational tracheostomy.

## Competing interests

The authors declare that they have no competing interests.

## Authors' contributions

MS, MM and SK have made substantial contributions to conception and design of the study as well as to the acquisition, analysis and interpretation of data. SB and KP have made substantial contributions to analysis and interpretation of data. MS, MM and SK have drafted the submitted manuscript. SB and KP have revised it critically for important intellectual content. All authors read and approved the final manuscript.

## Supplementary Material

Additional file 1 Number of publications reporting PDT-related fatalities and total number of publications on PDT procedures. Click here for file
