# Supplementary figures and images for: Death after percutaneous dilatational tracheostomy: a systematic review and analysis of risk factors
Source: Crit Care. 2013 Oct 29;17(5):R258. doi: 10.1186/cc13085 (PMC4056379; doi:10.1186/cc13085)

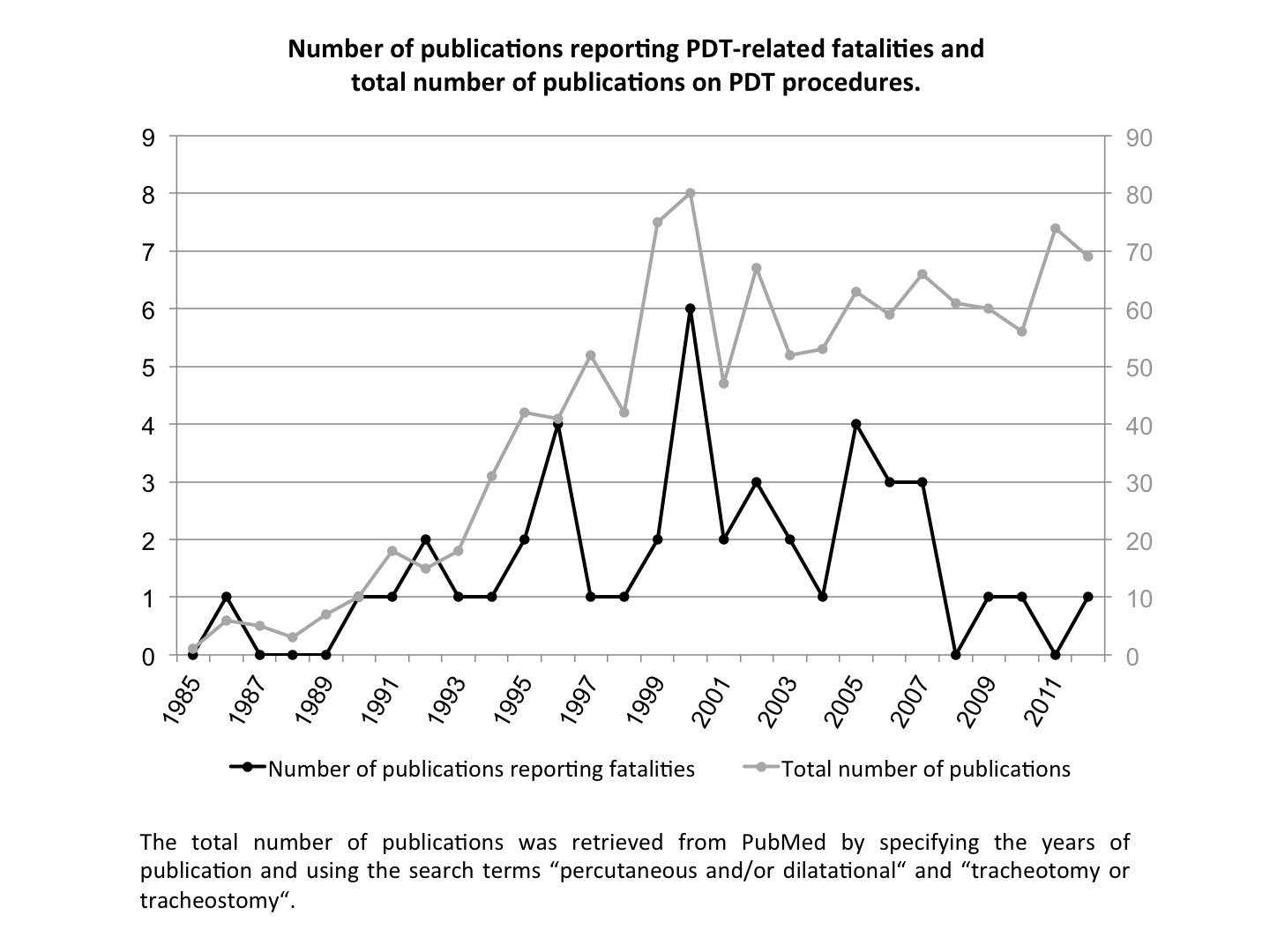

Supplement: Additional file 1 — Number of publications reporting PDT-related fatalities and total number of publications on PDT procedures. [file cc13085-S1.tiff]
